# Integrating artificial intelligence (AI) into colorectal cancer reporting

**DOI:** 10.1002/path.70029

**Published:** 2026-01-26

**Authors:** Konstantin Bräutigam, Ann‐Marie Baker, Viktor H Koelzer, Jakob N Kather, Trevor A Graham

**Affiliations:** ^1^ Centre for Evolution and Cancer Institute of Cancer Research London UK; ^2^ Institute of Medical Genetics and Pathology, University Hospital Basel University of Basel Basel Switzerland; ^3^ Computational and Translational Pathology Group, Department of Biomedical Engineering University of Basel Basel Switzerland; ^4^ Else Kroener Fresenius Center for Digital Health, Faculty of Medicine and University Hospital Carl Gustav Carus TUD Dresden University of Technology Dresden Germany; ^5^ Department of Medicine I, Faculty of Medicine and University Hospital Carl Gustav Carus TUD Dresden University of Technology Dresden Germany; ^6^ Medical Oncology, National Center for Tumor Diseases (NCT) University Hospital Heidelberg Heidelberg Germany; ^7^ Pathology & Data Analytics, Leeds Institute of Medical Research at St James's University of Leeds Leeds UK

**Keywords:** artificial intelligence, prognosis, colorectal cancer, cancer reporting, deep learning, prediction, evolution

## Abstract

Artificial intelligence (AI) and deep learning (DL) are transforming cancer research and clinical care, with histopathology playing a central role in this transformation. In colorectal cancer (CRC), the second leading cause of cancer mortality world‐wide, multimodal and vision‐language models (VLMs) hold particular promise for enhancing the standardisation of histopathology reporting, the understanding of disease biology, and the discovery of novel prognostic indicators. Despite the availability of guidelines and reporting templates for essential prognostic indicators, variability remains in how key features such as TNM staging or tumour deposits are assessed and reported in routine clinical practice. AI‐based tools have the potential to support refined extraction of established and extended features directly from whole‐slide images. In parallel, recent studies have shown that DL models applied to pathology slides and associated AI‐based biomarkers can outperform traditional histopathological prognostic indicators and uncover novel parameters, including tumour‐adipocyte interactions, tumour‐stroma ratio, and immune cell patterns at the invasive margin. Here, we review recent advances in both domains: AI‐assisted standardisation of CRC pathology reporting and AI‐driven identification of novel prognostic biomarkers. We highlight the need to refine and standardise CRC reporting practices and propose that a harmonised approach combining established pathology features with AI‐derived prognostic indicators could refine risk assessment and improve outcomes for CRC patients. © 2026 The Author(s). *The Journal of Pathology* published by John Wiley & Sons Ltd on behalf of The Pathological Society of Great Britain and Ireland.

## Introduction

Artificial intelligence (AI)‐related research in pathology has seen considerable progress in the last few decades [1]. Increased numbers of publicly available genomic and histological datasets, for instance The Cancer Genome Atlas (TCGA), have provided a valuable resource for expediting AI efforts and modelling [2, 3]. Recent developments include generic self‐supervised models for tissue classification and disease subtyping [4], foundation models [5, 6] for pan‐cancer detection, and large language models (LLMs) such as PathChat, a ChatGPT‐like [7] AI‐assistant for histopathology [8]. Diagnostic AI algorithms could improve turnaround times and pathology laboratory workflows [9], for example by faster case retrieval [10, 11]. They have also reached a remarkable level of accuracy in disease assessment [12]. Multiple studies have proven accurate disease classification using deep learning (DL), a subset of machine learning that uses advanced methods, such as artificial neural networks, to extract information [13, 14]. Furthermore, DL has demonstrated predictive power in various cancer types [15, 16, 17], for example aiding in the prognostic stratification of cancer patients [18, 19, 20, 21], supporting tumour content estimation for downstream molecular analysis [22], and extracting (novel) prognostic and/or predictive variables [23, 24]. Biomarkers are measurable indicators of biological states [25] and thereby can inform on disease state and progression.

In this work, we focus on the prognostic power of DL, i.e. AI‐based biomarkers, in colorectal cancer (CRC), the second most common cause of cancer‐related deaths in the world [26]. AI algorithms are capable of predicting outcomes in CRC patients more accurately than conventional histopathological standard variables such as tumour grade or vascular invasion [27, 28]. AI can predict a variety of factors in CRC, e.g. prognosis [28], treatment response [17], and tumour recurrence [29] (also for liver metastases [30]), and can accurately read microsatellite instability [31, 32, 33, 34, 35] and CRC consensus molecular subtypes (CMSs) [36] from slides. In addition, DL systems (DLSs) are safe to use in the biopsy context [37], distinguish pseudo‐ from true‐invasion [38], and classify specimens as typical, non‐neoplastic (e.g. reactive change), neoplastic [31], or simply as low‐ versus high‐risk [39, 40].

In principle, AI models can be categorised into explainable, partially explainable, and ‘black box’ models, depending on the extent to which their decision‐making process can be understood and interpreted by human experts (a short glossary is provided in Table 1).

**Table 1:** Short overview of AI learning approaches.

Term	Description
Explainable AI	AI models that offer insight into which features or patterns drive their predictions. These models aim for transparency and human interpretability, e.g. [41, 42, 43].
Black box model	Typically refers to complex, often unsupervised, models whose internal decision‐making processes are not transparent or easily interpretable, e.g. [13].
Unsupervised learning	AI models that derive structure or patterns from data without predefined labels or guidance, constructing an intrinsic ‘ground truth’ independently, e.g. [44].
Supervised learning	AI models trained using labelled data provided by an external source (e.g. expert annotations), offering a defined ground truth for model training, e.g. [31, 45, 46].
Self‐supervised learning	AI models that learn to extract discriminative features from input data (e.g. image tiles) without human‐provided labels, using internal learning signals, e.g. [34, 47].
Foundation model	Large‐scale AI models trained on broad and diverse datasets that can be adapted to a wide range of downstream tasks with minimal fine‐tuning, e.g. [4, 5].

For CRC, self‐supervised learning has already derived novel prognostic features hidden in morphology, such as the tumour adipose feature (TAF) [48], which was then translated into the histological parameter Stroma AReactive Invasion Front Areas (SARIFA), i.e. tumour cells in direct contact with adipocytes near the invasive front [49, 50]. AI reveals cancer features missed by pathologists, suggesting it sees beyond the human eye. This raises the question of revising cancer reports to include AI‐based biomarkers.

## Current CRC reporting in histopathology

Although internationally accepted guidelines are available, e.g. from the College of American Pathologists (CAP) [51, 52], the International Collaboration on Cancer Reporting (ICCR) [53], or in written form from the Royal College of Pathologists [54], cancer reporting is still not standardised across pathology institutions, with a significant proportion of pathologists not adhering strictly to reporting guidelines [55, 56]. It has been clearly demonstrated that structured reporting improves patient prognosis [57, 58, 59] and results in more comprehensive cancer reports [60]. Most institutions seem to report local (T‐stage) and nodal invasion, with lesser consistency in reporting, for instance, tumour grade and vascular invasion [61]. Peritumoural inflammation [62], tumour budding [63], and numerous other known prognosticators are reported heterogeneously. Cancer reporting is increasingly moving towards a synoptic format [61], meaning that reports are standardised [57, 64] and structured and include mandatory elements [65, 66]. This can involve the use of publicly available templates or, in the future, fully AI‐generated reports directly from tissue, as envisioned in approaches like HistoGPT [67]. Synoptic cancer reporting is associated with higher levels of satisfaction among clinicians [68]. LLMs have also demonstrated high accuracy in converting unstructured pathology reports to a synoptic format [69, 70], automatically generating synoptic reports [70] and retrieving accurate cancer staging information from narrative reports for multiple cancer types, including CRC [71]. Despite advances in structured reporting, CRC reports still focus primarily on a defined set of core elements (latest CAP version 4.4.0.0 June 2025) and include TNM stage [72], (lympho‐)vascular and perineural invasion, tumour grade, surgical procedure, specimen size, and resection margins. The most novel histopathological risk variables included are tumour deposits (incorporated into the seventh edition of the American Joint Committee on Cancer Staging Manual in 2010 [73]) and tumour budding [74, 75]. Prognostic genetic markers and molecular alterations, such as microsatellite status, are reported in a separate CAP protocol for biomarkers [76]. Overall, there is a clear need for integrated and standardised reporting of standard data elements with more novel biomarkers that can predict patient outcomes and treatment responses [77, 78].

## Histopathology features that predict prognosis of CRC

**Figure 1:**
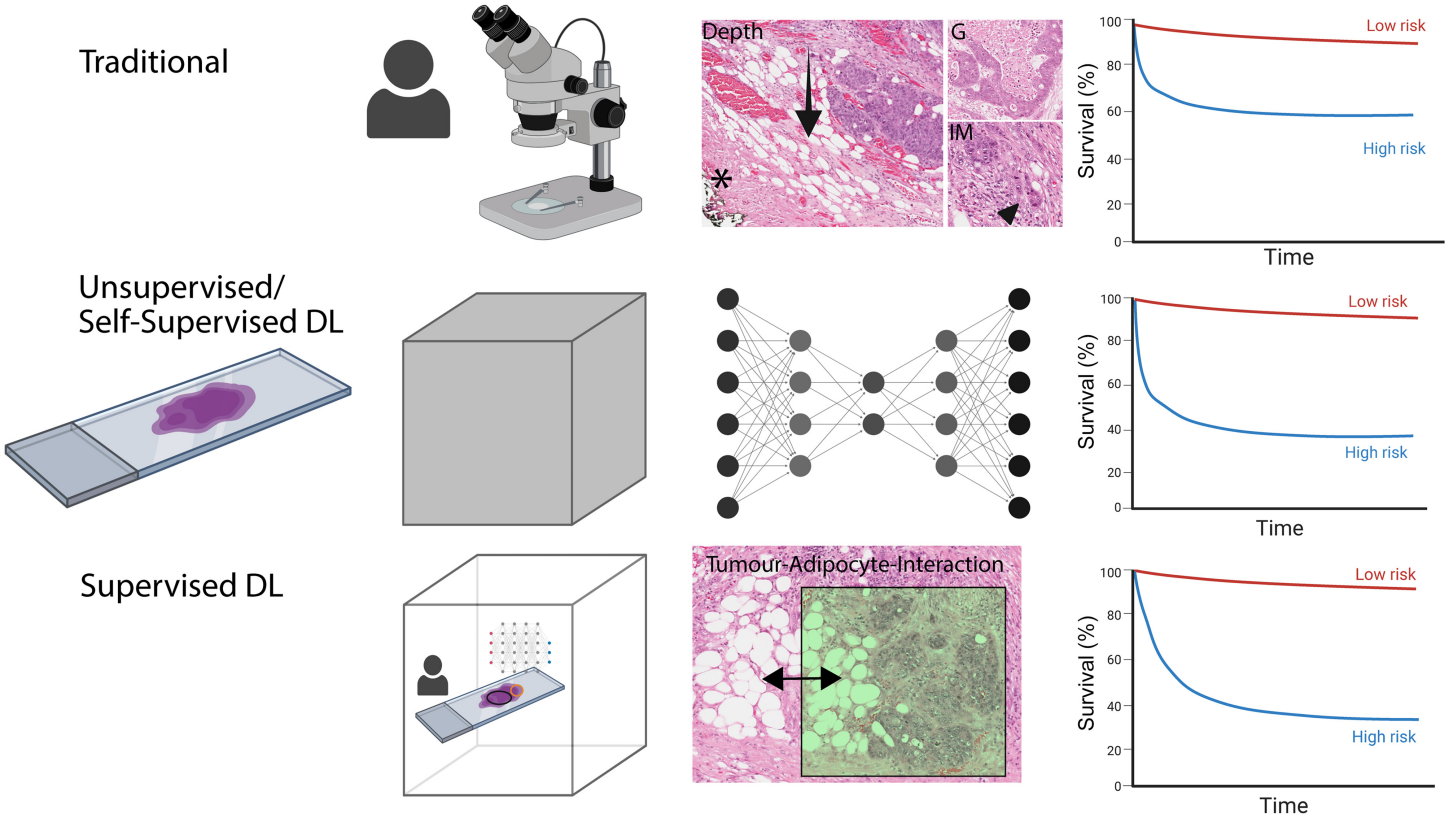
Conventional versus AI‐based cancer prognosis prediction and outcome. DL outperforms many conventional histopathological outcome predictors. Traditional prognostic predictors such as TNM stage [e.g. T stage by depth of invasion (arrow)], tumour grade (G) and invasive margin (IM, arrowhead: single cell infiltration), derived from human, microscopic histopathological assessment of a H&E‐stained slide (colorectal cancer (‘COAD’) from TCGA (*TCGA‐5 M‐AAT4*). Unsupervised or self‐supervised DL predicts patient prognosis on its own, leaving a ‘black box’ between the input and output of the algorithm. Supervised DL on whole slides, with regions of interest (highlighted in green) annotated by a human‐in‐the‐loop. Supervised DL‐based prognostication and traditional histopathologist‐driven prognostic assessments are labour‐intensive, requiring substantial human resources for data annotation, model training, and expert review. *Asterisk*: Deep margin. *Double arrow*: Tumour cell–adipocyte interaction at invasive margin of CRC. Created with Biorender.com. Bräutigam, K. (2025) https://BioRender.com/y28w7me.

**Figure 2:**
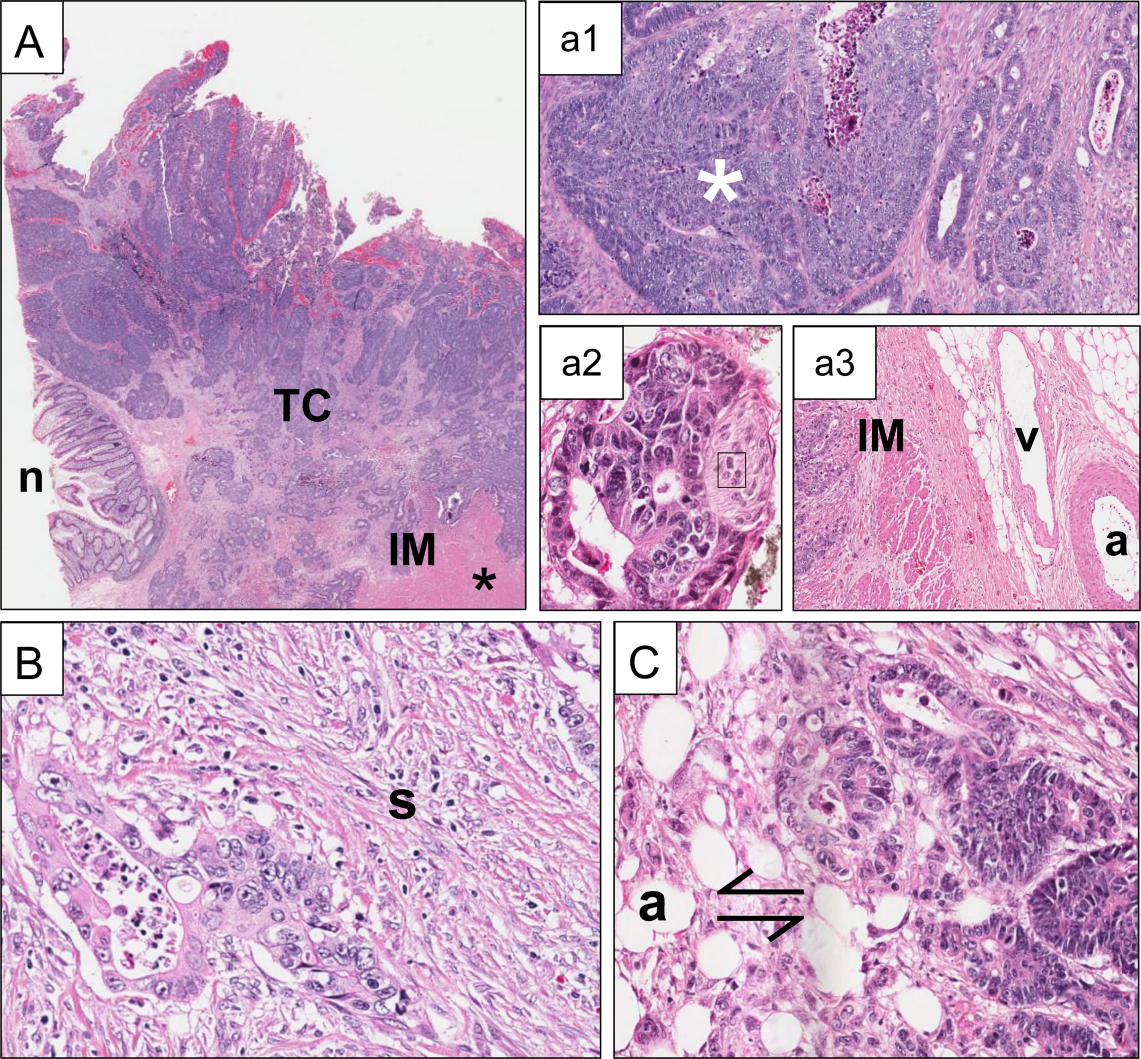
Histomorphological correlates of conventional versus DL‐derived prognostic predictors in CRC, H&E. (A) Overview of CRC sample (*TCGA‐5M‐AAT5*) demonstrating tumour centre (TC), invasive margin (IM), and adjacent normal mucosa (n). Traditional prognostic parameters such as depth of invasion (here pT3) and tumour grade (here G2) can be read from the slide. Both solid (white asterisk) and glandular growth patterns can be appreciated (a1). While there is perineural invasion (‘Pn1’, a2), blood vessels [artery (a), vein (v)] are not infiltrated (a3). (B) DL suggests prognostic significance of tumour immune microenvironment (TIME) and stroma (s). This tumour (*TCGA‐5M‐AATE*) is infiltrated by few tumour‐infiltrating lymphocytes (TILs) and has moderate desmoplastic stromal reaction. (C) The prognostic relevance of the interaction of cancer cells at the IM with adipocytes (a) – termed the TAF or SARIFA – has been revealed and confirmed by multiple studies (Table 1).

TNM staging is the gold standard for the prognostic F1 stratification of CRC (Figures 1, 2A). Both the number of positive lymph nodes (N‐stage) and count of harvested lymph nodes [79] and the positive‐to‐negative ratio [80] are prognostic factors. Metastatic disease, in line with real‐world pan‐cancer data (*n* = 15,726 patients) using explainable AI [41], remains a strong predictor of short survival. Clear surgical resection margins [81, 82] and vascular [83, 84] and perineural invasion [85] are important prognostic factors recognised by the Union Internationale Contre le Cancer (UICC), with TNM providing additional information beyond standard staging. Current CRC tumour grading, measured by the extent of gland formation (low versus high, based on the least differentiated component) [86], and tumour deposits (non‐continuous tumour nodules located within the colorectal mesentery, with no identifiable lymphatic, vascular, or neural structures present) are rather controversial prognostic factors [87, 88, 89] due to their subjective assessment and thereby weak to moderate interobserver reproducibility [90, 91].

TNM staging itself remains disputed in CRC [92], with good arguments for making revisions, such as the paradox of worse survival of Stage IIB/C versus Stage IIIA CRC patients – likely driven by an aggressive Stage II subset [93], and the greater importance of N‐stage in pT1 to pT3 tumours [94]. In line with that, there is evidence for the utility of a histological sub‐stratification of the depth of invasion in pT3 CRC [95]. Tumour buds [74] and poorly differentiated clusters are in general widely accepted by the histopathology community and can be scored reproducibly in H&E‐stained sections [75, 96]; however, some scepticism over their reliability as prognostic markers remains due to their at least partly questionable biology [97]. Further prognostic significance has been attributed to the tumour‐stroma ratio (TSR) in CRC [98] as in the Glasgow Microenvironment Score [99], and it can be measured using fully automated methods [100]. The Immunoscore (IS), which combines the densities of CD3^+^ and cytotoxic CD8^+^ T cells in the tumour and the invasive margin, has been validated as a predictor of CRC recurrence [101]. It has also been proven to be a prognostic factor for Stage III CRC patients with adjuvant treatment [102]. While the ESMO guideline committee recently recommended that the IS could be considered as part of the risk assessment for early‐stage CRC [103], neither the CAP nor the ICCR guidelines require the explicit implementation of the tumour immune microenvironment (TIME) in their CRC reporting templates, despite the evidence of its prognostic value in the form of the quantification and density of immune infiltrates [62, 101].

Some prognostic histomorphological features currently not incorporated as mandatory elements of standardised CRC reporting are summarised in Table 2.

**Table 2:** A selection of prognostic histomorphological features currently not incorporated as mandatory elements of standardised CRC reporting but readily quantified on routine H&E‐stained slides using AI automata.

	Human‐interpretable feature	Reporting	AI‐based quantification
Immune microenvironment	TILs	Counts, tiered scoring, subtyping (e.g. CD4^+^, CD8^+^, FOXP3^+^ T cells)	For example, in [104, 105, 106, 107]
	Degree of peritumoral infiltration	Pattern of inflammation, tiered scoring	
	(Tumour‐associated) Macrophages	Absolute counts, density, (cancer) proximity	For example, in [108, 109]
Stroma	Stromal proportion/percentage	E.g. Tumour‐stroma ratio	For example, in [100]
	Cancer‐associated Fibroblasts (‘CAFs’)	Absolute counts, density, (cancer) proximity	For example, [108]
	Tumour‐adipocyte interaction	Presence/absence of direct tumour‐adipocyte contact	For example, tumour‐adipose feature (‘TAF’) [110]
Cellular morphology/geometry	Cell shape (e.g. EMT spectrum)	Cytoplasmic radius, nuclear roundness	For example, ‘MorphoMIL’ [111]
	Micronuclei	Presence/absence	For example, ‘micronuclAI’ [112]
	Mitoses	Mitotic count per (tumour) area	For example, Optimised Mitoses Generator Network (OMG‐Net) [113], see also *MIDOG* [114]

Abbreviations: EMT: epithelial mesenchymal transition; *MIDOG*: mitosis domain generalization.

## Is it time to revise CRC reporting?

The need to revise CRC reporting to improve prognostic stratification has long been recognised. Weaknesses in current reporting practice include subjective tumour grading of CRC, often ambiguous depth of invasion (T stage) for malignant polyps, difficulties in detection of true vascular invasion and peritoneal involvement (pT4 stage), and the lack of systematic inclusion of molecular variables [115]. Also, resection margins and (lympho‐)vascular and perineural invasion are underreported in CRC [116, 117, 118, 119, 120]. Interestingly, hazard ratios (HRs) for clear resection margins in CRC are comparatively low (supplementary material, Table S1).

Although DL readouts could confirm the prognostic value (supplementary material, Table S1) of histopathological variables previously identified by humans (such as TNM or vascular invasion), markers discovered by AI appear to be effective at classifying CRC cases into distinct risk groups, as measured by HR (Table 3 and supplementary material, Table S1). AI markers can be combinations or refinements of human‐identified ones, such as the TSR [100, 121], but also novel markers such as TIME components, e.g. immune infiltrate, tumour‐adjacent stromal cells, and matrix [122, 123]. The prognostic, yet not predictive, relevance of tumour‐infiltrating lymphocytes (TILs, lymphocytes that have migrated into tumours) in the context of immunotherapy and checkpoint inhibition respectively (as approved for mismatch‐repair‐deficient rectal cancer [124]) has been recognised for years [62]. Evidence is accumulating for the prognostic relevance of the immune infiltrate, mainly CD8^+^ cytotoxic T cells, but also FOXP3^+^ T‐regulatory cells [125, 126, 127] and macrophages [108, 128]. TILs can be quantified reliably [104, 105, 129] and subtyped [106] by AI. Lower TIL counts have been significantly associated with shorter overall survival (OS) [129] and recurrence in CRC [105]. In addition to this, we are aware of the importance of the invasive margin of CRC as a site of pronounced tumour–immune interaction [130] with higher immune surveillance [131]. To date, this ‘spatial’ dimension of tissue‐derived cancer biomarkers is not included in CRC reporting.

**Table 3:** DL models predicting CRC prognosis. The table summarises network type, cohort size, endpoints, and potential novel predictor variables or human‐interpretable histologic features respectively.

Authors	Journal, year	Network	Cohort/sample size	Endpoint	Novel ‘feature’	Prognosis
Skrede *et al* * [28]	*The Lancet*, 2020	Ten CNNs (‘DoMore v1’ networks) with predefined image tiles from H&E WSI, multiple instance learning	Training cohort *n* = 828 patients, test cohort *n* = 920 patients, validation cohort *n* = 1,122 patients (Stage II and III CRC)	Cancer‐specific survival (‘DoMore‐v1‐CRC’ marker)	No, ‘unknown biological correlate’, automatic prediction	Classifier outperforms most other markers, e.g. T stage, lymphovascular invasion, in stage‐specific multivariable analyses
Foersch *et al* [17]	*Nature Medicine*, 2022	Multistain deep learning model (one individual 18‐layer residual neural network) on WSI using CD4, CD8, CD20, CD68	Four pan‐stage CRC cohorts with *n* > 1,000 patients in total (neoadjuvant and prognostic cohorts)	RFS (using AImmunoscore (AIS)‐low, −intermediate and −high) and response prediction to neoadjuvant radiochemotherapy (rectal cancer)	Yes, multi‐parametric TIME (‘explainable’ AI), tumour cell–adipocyte co‐localisation	AIS outperforms IS, UICC stage, and resection status
Wulczyn *et al* † [110]	*npj Digital Medicine*, 2021	Weakly supervised deep learning system (DLS)/CNN on H&E WSI (tumour segmentation model combined with prognostic model)	*n* = 3,652 stage II and III CRC patients, two validation datasets with *n* = 1,239 and *n* = 738 cases	DSS	Yes, poorly differentiated tumour cell clusters adjacent to adipose tissue	DLS outperforms conventional predictors such as venous invasion
Bychkov *et al* [20]	*Scientific Reports*, 2018	Pretrained VGG‐16 CNN and recurrent neural network (long short‐term memory; LSTM), H&E TMA (core diameter: 1 mm)	*n* = 420 CRC patients, dichotomised into a low‐ and high‐risk group	Five‐year DSS	No, ‘exploratory’ analysis	Network predicts survival better than tumour grade, visual assessment by a pathologist, and Dukes' stage
Kather *et al* [27]	*PLoS Medicine*, 2019	Deep CNN pretrained on the ImageNet database, H&E WSI	Independent, multicentre CRC cohorts including *n* = 1,382 (86 + 25 + 862 + 409) WSI	OS	Yes, Deep Stromal Score (DSS)	DSS is an independent prognostic factor for OS
Höhn *et al* [19]	*npj Precision Oncology*, 2023	DL pipeline with tissue subtyping (nine colorectal tissue type classes), feature extraction, tile‐to‐tile aggregation and network	Pan‐stage CRC, training cohort *n* = 2,205 patients, four test cohorts *n* = 2,866 (545 + 1,340 + 610 + 371) patients	Five‐year survival curve (predicting monthly hazards)	No	Survival curve more nuanced but did not outperform binary classification (low versus high risk)
Zhao *et al* [132]	*EBioMedicine*, 2020	CNN (Visual Geometry Group 19‐layer model) with transfer learning for fully automated TSR quantification on H&E WSI	Two independent sets, discovery cohort *n* = 499 patients (stage I–IV), validation cohort *n* = 315 patients (stages II and III CRC)	OS	TSR	Stroma‐high is associated with reduced OS in both cohorts
Sun *et al* [133]	*Computer Methods and Programs in Biomedicine*, 2022	Unsupervised multiple instance learning model on H&E WSI (variational autoencoder and generative adversarial network)	Training cohort *n* = 147 patients, validation cohort *n* = 63 patients (both cohorts stage III CRC)	DFS and OS	No	Outperforms clinicopathological characteristics in predicting DFS and OS

* Follow‐up study by Kleppe *et al* [18].

^†^ Validation by L'Imperio *et al* [134].

Abbreviations: CD, cluster of differentiation; DFS, disease‐free survival; DLS, deep learning system; DSS, disease‐specific survival; IS, Immunoscore; RFS, relapse‐free survival; TMA, tissue microarray; UICC, Union Internationale Contre le Cancer; WSI, whole‐slide image.

Apart from the immune infiltrate, it seems that pathologists neglect the prognostic relevance of the non‐tumour compartment (cellular and non‐cellular/stromal, terminology explained in more depth elsewhere [135]) in CRC reporting. The deep stroma score – a DL‐derived parameter summarising the prognostic power of various non‐tumour components such as desmoplastic stroma, lymphocytes, and adipose tissue – predicts the outcome in CRC [27]. This is supported by mouse models that highlight poor outcomes associated with TGF‐β‐driven stromal infiltration in CRC [136, 137], as well as the CMS, which are categorised by gene expression programmes. Of particular note is the CMS4 subtype, which exhibits mesenchymal characteristics and stromal invasion [138]. The CMS4 subtype is also associated with pronounced epithelial‐mesenchymal transition (EMT), which drives tumour aggressiveness and deteriorates drug response in CRC [139]. An elongated cell phenotype with a modified cytoskeleton [140] is often the histomorphological EMT correlate, which can be well quantified using AI automata [141]. Apart from cellular morphology, LLMs are able to capture EMT states from single‐cell transcriptomics [142]. The fact that DL identifies cancer cells interacting with adipocytes at deep invasion as prognostic re‐confirms the importance of single cells at the invasive margin and adds an environmental factor (adipose tissue) to it [143].

## DL can outperform conventional histopathologic outcome predictors

Over the past decade, multiple DL algorithms have shown impressive prognostic performance in large digitised CRC cohorts (Table 3 and supplementary material, Table S1). Most of these DL models are based on H&E‐stained slides [144]. The features which these AI algorithms ‘see’ that provide prognostic value are sometimes elusive (a black box), although feature‐extraction algorithms are able to reveal novel or re‐confirm conventional prognostic variables (Figure 1). For instance, a large foundation model [5] attributed high levels of attention to fibrosis in lung adenocarcinoma, to tumour budding in lung squamous cell carcinoma, and to necrosis in both lung cancer subtypes.

Already in 1997, a neural network fed with multiple clinical variables outperformed population statistics in the prediction of patient death in CRC [145]. Using large training, tuning, test, and validation cohorts, Skrede *et al* [28] demonstrated the prognostic superiority of their convolutional neural network (CNN) over standard variables such as pT2 stage or lymphovascular invasion in Stage II and III CRC (supplementary material, Table S1). The biological proxy of their network was, however, unknown. In a follow‐up study, their DoMore‐v1‐CRC marker, combined with existing prognostic variables, was successfully translated into a clinical decision support system and ultimately proved to be a (more) reliable indication for adjuvant treatment of Stage II and III CRC [18]. Another study showed that AI outperformed the visual scoring of three pathologists in tumour grading [20], while another unsupervised multiple‐instance model surpassed clinicopathological characteristics for Stage III CRC prognosis [133]. However, this was limited by a rather small validation cohort (Table 2). Both studies were exploratory and did not derive human‐interpretable features. Furthermore, they lacked long‐term validation in multiple centres. In a head‐to‐head comparison with the main prognostic predictors, i.e. TNM stage, DL models showed a higher HR than the pT2 and pT3 stages, but not the pT4 stage [28, 110, 133]. Looking at all included studies (supplementary material, Table S1), the highest HRs were observed for the N2 (*p* < 0.0001 [28]) and pT4 stages (*p* < 0.001 [110]), which highlights the power of the TNM system in advanced disease.

In addition to the black box models, explainable AI suggests that TIME features are hidden prognosticators (Figure 2B), particularly when subsets of different immune cells (e.g. CD4^+^, CD8^+^, CD20^+^, and CD68^+^) are used to cover a large immune microenvironment [17, 127]. The AImmunoscore (AIS) outperformed clinically relevant prognostic parameters including the IS [101, 146], UICC stage, and resection (R) status [17]. Similarly, a weakly supervised DLS was able to outperform TNM stage and venous invasion in terms of HR (supplementary material, Table S1) in CRC [110]. Interestingly, their clustering‐derived feature extraction revealed tumour cell interaction with adipose tissue (Figure 2C) as a prognostic factor. The interaction of tumour cells with adipose tissue was confirmed by attention‐based self‐supervised DL in CRC (*n* = 4,428 patients) [147]. Unfortunately, so far, the potency of novel morphological features has not been compared against conventional (and AI) biomarkers in multivariate analyses.

A low TSR has been extracted as a high‐risk feature, having been previously shown to be prognostic for OS in CRC using a fully automated DL algorithm [132]. However, in terms of HR, the ‘stroma‐high’ subset (HR 2.08; *p* = 0.004) was still inferior to Stage III (HR 2.98; *p* < 0.001). Ploidy and stromal‐epithelial ratio can synergistically stratify Stage II CRC patients into distinct mortality risk groups [148]. Perhaps surprisingly, the presence of TILs and desmoplasia has not been attributed high attention in other DLSs [110]. Showing that complex cell decomposition and TIME segmentation is possible, the DL‐derived deep stroma score has been shown to be a strong survival prognosticator in CRC [27]. However, it was not possible to determine which component of the non‐tumour compartment was (most) prognostic and how it compared to conventional predictors. Lastly, published DL algorithms have demonstrated robust performance in predicting lymph node metastasis in CRC, influencing N‐stage, which has a critical impact on patient prognosis [149, 150]. Moving away from traditional tumour grading, AI‐based single‐cell analysis has stressed the importance of stromal cells as a prognostic parameter [107]. Last but not least, tumour bud assessment has been shown to be consistently performed fully automatically using AI [151].

In light of the foregoing discussion, we propose a concise CRC reporting protocol that includes so‐called traditional CRC reporting items with proven prognostic value, alongside novel (AI‐based/driven) parameters (microenvironmental, spatially resolved, and molecular; Table 4). We advocate a strictly evidence‐based approach to cancer reporting that includes items which have been robustly validated in multiple international cohorts, ideally prospectively. To avoid diluting the report, we recommend presenting the most prognostic variables first. Descriptive parameters and ‘gross items’ are listed at the bottom, as they mainly serve the purpose of quality control (e.g. surgical specimen dimensions) and should not mask the main variables. Kleppe *et al* have led the way in this area by validating their AI biomarker in over 2,000 Stage II/III CRC patients within their clinical decision support system [18, 28]. In lung cancer, others recently tested their H&E‐based EGFR‐mutation detection algorithm in a ‘silent trial’ using real‐world data in real time [152]. Cancer reporting items should be continually re‐evaluated against new prognostic factors in a multivariate, inclusive framework considering gender and ethnicity, followed by meta‐analysis, refinement, and report updates.

**Table 4:** Vision of mandatory elements of colorectal cancer report of the future. Most prognostic items should be reported first in bold. Gross items and other less significant prognostic factors should not distract from the key variables and are therefore listed at the bottom.

Reporting item	Comment
**TNM stage + R status**	High T‐stage, nodal, and distant metastasis are highly prognostic, resection status (i.e. margin) an indicator of local recurrence
**TIME composition** Stromal proportion, e.g. TSR Immune infiltration (with at least basic deconvolution into CD4^+^, CD8^+^, CD20^+^, CD68^+^ and FOXP3^+^), e.g. binary (high versus low) or fully automated per ROI	Offers strong prognostic utility, augmented by spatially resolved tissue features and precise digital quantification at cell level
Vascular and perineural invasion, tumour grading, histological subtype, tumour budding, and tumour deposits	Established additional prognostic indicators
**Molecular data** MSI and BRAF V600E testing KRAS status (and potentially other EGFR pathway genes) ctDNA	Predictive (e.g. response to EGFR blockade) and prognostic, detection of MRD
Macroscopic assessment (e.g. procedure, tumour site, tumour dimensions, perforation)	Complementary to clinical information/quality control

Abbreviations: ctDNA, circulating tumour DNA; MSI, microsatellite instability; Pn, perineural invasion; R status, resection status; ROI, region of interest; TIME, tumour immune microenvironment; TSR, tumour‐stroma ratio.

## Morphology of CRC evolution

Histomorphology can also be used as a proxy for cancer evolution. Chromosomal instability (CIN) and intratumoural heterogeneity (ITH) are well‐known features of CRC evolution. In particular, ITH – in terms of cancer cell morphology [45] – can be well quantified using DL, as objective quantification is impractical for the human eye. CIN‐associated histomorphology corresponds to cytological abnormalities such as nuclear budding or micronuclei, which are quantifiable by automated methods [112].

**Figure 3:**
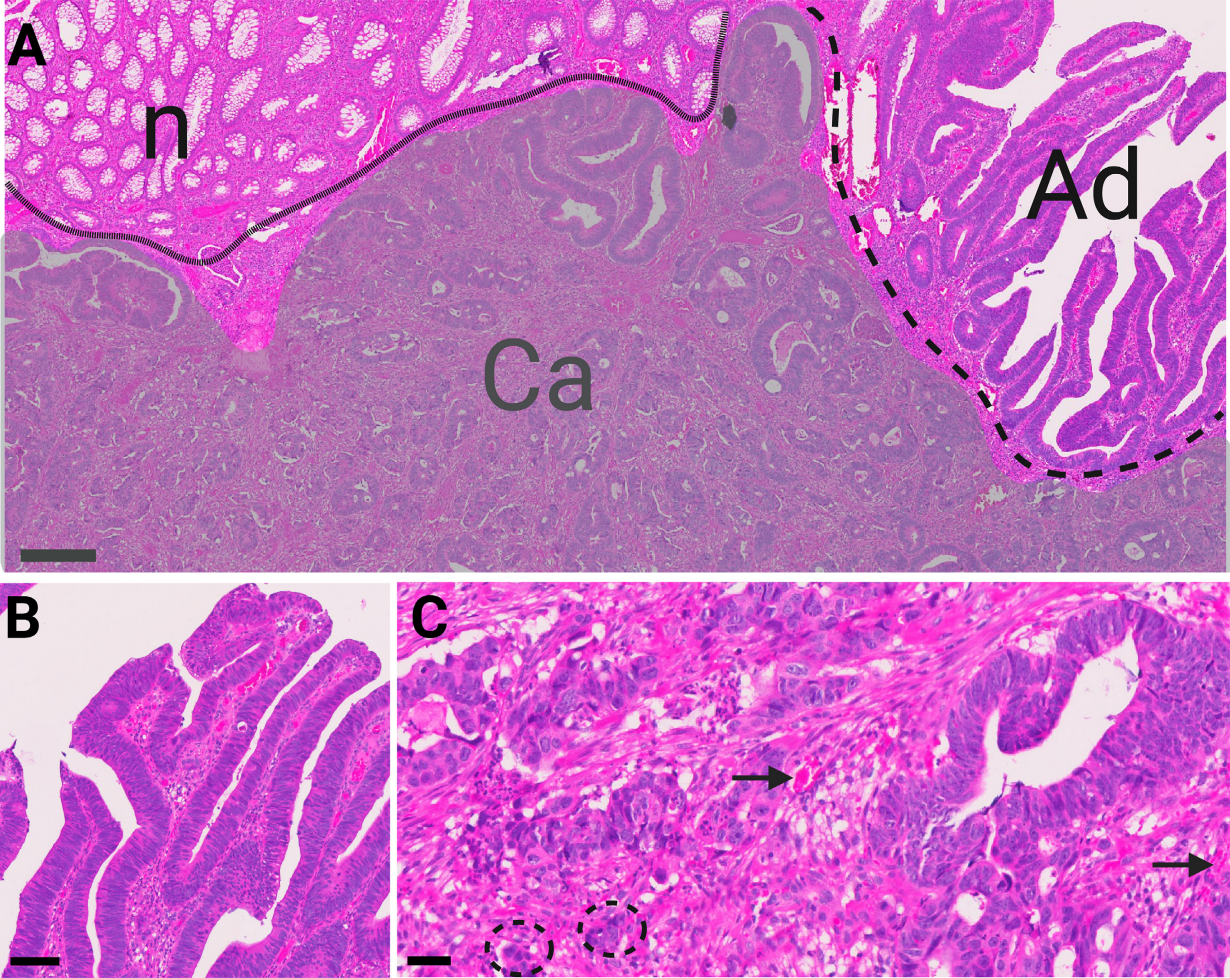
The morphology of CRC evolution. (A) Increasing architectural complexity in adenomatous glands (Ad) versus healthy mucosa (n). The adenocarcinoma (Ca, grey shade) infiltrates deeply into the tissue, forms solid clusters, and grows vertically (in‐house CRC cohort). H&E, scale bar, 300 μm. (B) Architecturally complex adenomatous glands with a loss of surface maturation (goblet cell depletion) and sharp nuclear polarisation (pencillate nuclei), indicating a transformative cellular state. H&E, scale bar, 100 μm. (C) Moderately to poorly formed CRC glands, embedded in desmoplastic stroma with occasional microvessels (arrows) and sparse immune infiltration. Cell clusters and single cells detach from the adenocarcinoma glands (encircled, dashed), some showing a pointed contour indicating morphological plasticity and perhaps an EMT. H&E, scale bar, 40 μm.

Architectural features of (early) CRC evolution (Figure 3A) are crypt branching, crypt budding, and fissure and represent increasing biological complexity [153, 154]. During adenoma evolution, nuclear (pseudo‐)stratification and loss of surface maturation (with nuclear polarisation) are indicative of initial carcinogenesis and dedifferentiation (Figure 3B). Current tumour grading in CRC, i.e. an assessment of tumour differentiation, reflects the extent to which the tumour represents its original ‘lineage‐true’ appearance (gland‐forming). Increasing loss of differentiation correlates with a less controlled biological state, loosening inherent biological constraints and allowing more aggressive behaviour, i.e. from a well‐formed glandular morphology to poorly defined clusters or even single‐cell infiltration. Dedifferentiation aligns with digitally quantifiable metrics such as cellular shape (roundness and contours) and geometry [111], e.g. cytoplasmic radius. Dedifferentiation culminates in the invasive EMT phenotype, with cytoskeletal rearrangement towards a more spindle‐shaped, elongated cell body to enhance motility and migration [155]. Morphological ‘plasticity’ may be associated with more aggressive tumours, as a sign that cells are more able to adapt to new selective pressures. Ongoing CRC evolution is also shaped by microenvironmental cues. Tumour‐induced stromal recomposition (Figure 3C) and matrix remodelling in desmoplasia form a mechanical barrier, allowing an immune‐suppressed niche [156]. Environmental pressure such as hypoxia goes along with evolving clones stimulating angiogenesis, which can be digitally quantified as microvessel counts and vessel density [157].

## Why are AI predictions more accurate?

A precise answer here is challenging: many powerful AI algorithms are black boxes, where it is unclear what the algorithm is ‘seeing’. Nevertheless, what is clear is that AI algorithms can potentially ‘see everything’ there is to see. First, AI can integrate multiple types of data, combining histological features from H&E‐stained slides with other data modalities such as immunohistochemistry, genomic and transcriptomic profiles, spatial organisation of tissue compartments, and even clinical or radiological data. This multimodal integration enables AI systems to uncover complex relationships between tissue morphology and underlying tumour biology, potentially improving the accuracy of outcome prediction and enabling more personalised risk stratification, which remains inaccessible in current ‘slide‐based’ clinical pathology workflows. Second, it analyses and identifies complex and combinations of patterns, e.g. not only the stromal area but also stromal cell type composition, organisation, and the relationship with tumour cells. Third, ‘unsupervised’ AI algorithms use feature extraction‐based approaches to morphological parameters that correlate with outcomes (such as the TAF). Fourth, AI predictions are more scalable, faster, and reproducible and have demonstrated the ability to stratify patient risk with greater granularity than traditional histopathological assessments. Historically, most prognostic markers in pathology have been implemented as binary decisions (e.g. present versus absent) or coarse categorical classifiers, reflecting the human preference for discrete, easily actionable categories. In contrast, AI models operate on continuous probability distributions and multidimensional feature spaces, enabling a more refined and data‐driven assessment of disease risk that captures subtle gradations in tumour biology and patient prognosis. Similarly, we note that EAGLE, a recently published DL framework, can combine both image tiling and selection of relevant morphological features [158], thereby simulating the human pathologist in an efficient diagnostic process. It is an example of how AI algorithms can be constructed to ‘explain themselves’.

## Challenges and outlook

In summary, AI confirms conventional prognostic variables and reveals more complex histological patterns, e.g. ratios (TSR) instead of categorical scoring and precise cell‐to‐cell type interactions respectively, with clinical relevance in CRC [17]. Multiple DL studies on CRC prognostication highlight the prognostic potential of both explainable AI and black‐box DL models. However, due to the heterogeneity of the prognostic markers investigated, the inclusion of different UICC stages and different survival endpoints between those studies, it is not possible from published data to make a robust one‐to‐one comparison of their prognostic efficacy, especially as the ‘effect sizes’ of AI are not clearly defined. However, we believe that cancer reporting should include AI‐derived features, given their impressive prognostic potential, if HRs are used as a surrogate for effect size. We strongly advocate conducting prospective clinical trials that compare standard prognosticators with AI‐derived ones.

Both MSI and BRAF mutations are important prognostic and targetable alterations in CRC [159, 160]. As previously mentioned, DL can detect these molecular alterations [35, 161], but generalisability is limited by a lack of cohort size and diversity as well as inconsistency in the histomorphological features that predict the respective alteration (e.g. TILs for/in MSI) [162]. To accurately detect rarer mutations from a ‘slide’, more cohort diversity is needed [161]. The fact that most models do not incorporate biopsies limits the ability to make predictions in a pre‐ or non‐operative context [32, 163, 164]. Nevertheless, we consider the use of H&E‐based AI for detecting key mutations to be extremely valuable, given that developing countries have limited access to genomic facilities and sequencing [165]. In addition, circulating tumour DNA (ctDNA) has emerged as a powerful tool for risk stratification, for example in the detection of minimal residual disease (MRD) in the adjuvant setting [166, 167, 168]. Quantifying ctDNA is possible using DL [169] and combining DL with MRD has added prognostic value [21].

Overall, we believe that next‐generation sequencing (NGS) data should be incorporated into synoptic cancer reporting, with DL assisting in determining which variants should be reported [170]. MSI and *BRAF* testing, as well as *KRAS* status and EGFR pathway genes, is critical given their prognostic and predictive value (EGFR blockade) [167, 171].

Existing work on AI tools for pathology reports has mainly focused on DNA mismatch repair status, *BRAF* V600E, and pathologic N‐stage reporting with little inclusion of, for instance, tumour deposits, margin status, or histological subtypes [172]. Nevertheless, DL was shown to outperform many of the ‘classical’ prognosticators of poor outcome such as vascular invasion, tumour grade, and (clear) resection margins. Further, most of the novel prognosticators summarised here are applicable for both CRC biopsy and resection specimens, as both capture the immediate microenvironmental niche in cellular and morphological detail.

DL models combined with existing clinicopathological evidence support the need for ‘investigative pathology’ [173]. This means moving away from cancer ‘alone’ and towards the TIME. The deep stroma score or the TSR are attempts to do this [27, 100]. To further refine cancer prognosis, and to see and report what AI sees, we should demystify the black box. Firstly, this will need more explainable, multimodal AI models with a solid basis for human interpretation. Heatmap visualisation of regions of interest (ROIs) [46, 174] is an important step to support explainability (e.g. class activation mapping [175]). Second, more interaction with the ‘human‐in‐the‐loop’ [176] could be beneficial, for instance, as a means of diagnostic ‘fine‐tuning’ [177]. With regard to the global pathologist shortage [178], the automated, self‐supervised detection of ‘discriminatory’ histopathologic features in tiles [47] is an alternative. Third, the potential of DL to uncover novel and prognostic morphological aspects through feature extraction for better stratification of cancer patients should be exploited. For example, transformer networks for patch aggregation allow more effective modelling of complex relationships in histopathological images [35].

Implementing AI/DL algorithms is fraught with regulatory difficulties [179]. This stems from a complex legal landscape that has not yet adapted to dealing with (novel) AI‐based applications [180]. In 2021, Paige Prostate became the first AI‐based application to receive FDA approval [181], setting a precedent. The translational pipeline involves robust validation using large and diverse datasets, extensive testing, and precise risk–benefit calculations, as well as demonstrating clinical utility and validity [182]. Generally speaking, it appears that ‘explainable AI’ algorithms with a narrow focus and human‐interpretable features incur fewer regulatory constraints than black box models [183].

In addition to regulatory and financial hurdles [184], the implementation of AI in histopathology routines faces intrinsic morphology‐related challenges. Histomorphological CRC subtypes [185] might be left underdiagnosed and thereby incorrectly stratified in diagnostic algorithms. In addition, AI models are, at least up till now, mostly limited to sets of training cohorts, which might narrow their future learning capacity [186]. Interobserver variability prevails also for pT4 CRC, with histological criteria not universally accepted [187, 188] and serosal (outermost layer of the colon wall) involvement difficult to detect [189]. As commercial AI systems proliferate, establishing consensus on performance metrics has become essential (guidance on metrics for AI decision systems in [190]). AI‐derived misdiagnoses have evoked the fear of medical malpractice [191] and the associated question of who is legally liable. But despite the fear of ‘fragile algorithms’ [192], it is questionable if the black box is really unavoidable [193] or the extent to which the level of ‘opacity’ should be acceptable [194]. The question has been raised as to whether we should strive for explainability at all [195] or whether we should simply consider learning from the black box. The human interpretability of AI algorithms in cancer care is the subject of ongoing debate, with different perspectives emerging. We agree with the argument that, provided the black box consistently produces the best results, it should be considered. Human decision‐making can be equally opaque and may lack explainability and transparency [196, 197]. Furthermore, each DL ‘mode’ has context‐dependent advantages and disadvantages. For example, foundation models are very resource‐intensive but also highly scalable, whereas unsupervised learning is less accurate but label‐independent [198]. Ultimately, the implementation of DL algorithms necessitates robust computational infrastructure and expertise.

Overall, AI and DL allow us to review large datasets in a short time and detect previously unrecognised patterns with less interobserver variability. Additionally, AI can facilitate equitable access to expert pathology services by enabling centralised AI computing centres where images from various locations can be uploaded. This enables even rural or resource‐limited regions to benefit from state‐of‐the‐art pathology services [199]. Furthermore, a low‐cost workstation has been developed on which DL algorithms can achieve a similar level of accuracy, running entirely on open‐source software [165]. A wide range of open‐source software is available for slide annotation, tissue segmentation, and script implementation [200]. Additionally, a web‐based ‘return on investment’ calculator has been published for use in settings with limited resources [201]. Although digitisation may seem expensive at first, it can also reduce costs by eliminating the need for glass storage and enabling virtual slide review. Furthermore, generative AI has the potential to replace costly antibody panels [202].

As AI becomes more effective at predicting the prognosis of CRC patients, we should consider incorporating AI‐generated variables into our cancer reporting protocols, or at least revising and refining conventional risk parameters according to the models' suggestions. We believe that cancer reports should prioritise items with the best prognostic and predictive value. Incorporating novel AI‐based biomarkers will not replace pathologists, but it will increase the power of their reports.

## Author contributions statement

KB and TG conceived the manuscript, KB drafted it, and A‐MB, VHK, JNK and TAG edited it. All authors approved the final version.

## Supporting information


**Table S1.** HRs of conventional versus DL‐derived prognostic predictors in CRC (only multivariate analyses are included, ordered from highest to lowest HR per study)

## Data Availability

Data sharing is not applicable to this article as no datasets were generated or analysed during this study.
