# Supplementary material for: Integrating artificial intelligence (AI) into colorectal cancer reporting
Source: J Pathol. 2026 Jan 26;268(4):367–82. doi: 10.1002/path.70029 (PMC12984008; doi:10.1002/path.70029)
Supplement: Supplementary file 1 — Table S1. HRs of conventional versus DL‐derived prognostic predictors in CRC (only multivariate analyses are included, ordered from highest to lowest HR per study) [file PATH-268-367-s001.docx]

**Integrating artificial intelligence (AI) into colorectal cancer reporting**

K Bräutigam *et al. J Pathol* <https://doi.org/10.1002/path.70029>

**Supplementary Table S1**

**Table S1.** Hazard ratios (HRs) of conventional versus deep learning (DL)-derived prognostic predictors in colorectal cancer (CRC) (only multivariate analyses are included, ordered from highest to lowest HR per study).

| **Study** | **Subgroup** | **Marker** | **UICC** | ***P*-value** | **N_validation cohort** | **HR (95% CI)** |
| --- | --- | --- | --- | --- | --- | --- |
| Bychkov *et al* [20] |  | Age (≥ 75 yrs) | NA | < 0.001 | 60 | 3.41 (2.06–5.65) |
|  |  | Age (65–74 yrs) | NA | 0.008 | 60 | 1.91 (1.18–3.08) |
|  |  | LSTM | NA | < 0.001 | 60 | 1.89 (1.41–2.53) |
|  |  | Visual scoring | NA | 0.030 | 60 | 1.42 (1.05–1.94) |
| Foersch *et al* [17] |  | Immunoscore 2 | 1 to 4 | 0.037 | 59 | 2.19 (1.05–4.58) |
|  |  | UICC stage | 1 to 4 | < 0.001 | 59 | 1.97 (1.55–2.51) |
|  |  | L1, V1, Pn1 | 1 to 4 | 0.051 | 59 | 1.43 (0.99–2.06) |
|  |  | R status | 1 to 4 | 0.237 | 59 | 1.32 (0.8–2.16) |
|  |  | Immunoscore "Best" | 1 to 4 | 0.247 | 59 | 0.73 (0.43–1.24) |
|  |  | Immunoscore 3 | 1 to 4 | 0.267 | 59 | 0.71 (0.38–1.31) |
|  |  | AIS | 1 to 4 | < 0.001 | 59 | 0.5 (0.36–0.69) |
| Kather *et al* [27] |  | Deep stroma score | 1 to 4 | 0.003 | 409 | 1.99 (1.27–3.12) |
| Skrede *et al* [28] |  | pN2 | 2 and 3 | < 0.0001 | 1,122 | 5.94 (3.71–9.52) |
|  | Without adjustment* | DoMore-v1-CRC: poor prognosis | 2 and 3 | < 0.0001 | 1,122 | 3.84 (2.72–5.43) |
|  | With adjustment* | DoMore-v1-CRC: poor prognosis | 2 and 3 | < 0.0001 | 1,122 | 3.04 (2.07–4.47) |
|  |  | pT2 | 2 and 3 | 0.006 | 1,122 | 1.86 (0.9–3.86) |
|  |  | pN1 | 2 and 3 | < 0.0001 | 1,122 | 1.84 (1.13–2.98) |
|  |  | pT4 | 2 and 3 | 0.006 | 1,122 | 1.75 (0.9–3.86) |
|  |  | L1 | 2 and 3 | 0.023 | 1,122 | 1.66 (1.07–2.56) |
|  |  | V1 | 2 and 3 | 0.710 | 1,122 | 1.07 (0.76–2.56) |
| Sun *et al* [130] | OS | Substage | 3 | 0.063 | 63 | 2.13 (0.96–3.09) |
|  | DFS | Bowel obstruction | 3 | < 0.001 | 63 | 1.71 (1.28–2.29) |
|  | DFS | pN1 versus pN2 | 3 | 0.275 | 63 | 1.47 (0.73–2.96) |
|  | DFS | DLS | 3 | < 0.0001 | 63 | 1.31 (1.17–1.47) |
|  | DFS | Substage | 3 | 0.445 | 63 | 1.29 (0.67–2.49) |
|  | OS | pN1 versus pN2 | 3 | 0.579 | 63 | 1.28 (0.53–3.09) |
|  | OS | DLS | 3 | 0.017 | 63 | 1.19 (1.03–1.38) |
|  | OS | Adenoma | 3 | 0.156 | 63 | 0.74 (0.5–1.12) |
|  | DFS | Adenoma | 3 | < 0.001 | 63 | 0.58 (0.4–0.82) |
| Wulczyn *et al* [131] | Validation Set 2 | pT4 | 2 and 3 | < 0.001 | 738 | 4.25 (1.95–9.28) |
|  | Validation Set 2 | pT3 | 2 and 3 | 0.032 | 738 | 2.31 (1.07–4.98) |
|  | Validation Set 2 | pN2 | 2 and 3 | < 0.001 | 738 | 2.21 (1.58–3.08) |
|  | Validation Set 1 | pN2 | 2 and 3 | < 0.001 | 1,239 | 2.03 (1.55–2.67) |
|  | Validation Set 2 | G2 | 2 and 3 | 0.360 | 738 | 1.98 (0.92–4.25) |
|  | Validation Set 2 | G3 | 2 and 3 | 0.119 | 738 | 1.86 (0.85–4.07) |
|  | Validation Set 1 | pT4 | 2 and 3 | 0.037 | 1,239 | 1.66 (1.03–2.65) |
|  | Validation Set 1 | DLS risk score | 2 and 3 | < 0.001 | 1,239 | 1.54 (1.38–1.7) |
|  | Validation Set 2 | DLS risk score | 2 and 3 | < 0.001 | 738 | 1.42 (1.25–1.61) |
|  | Validation Set 2 | Age | 2 and 3 | < 0.001 | 738 | 1.31 (1.17–1.47) |
|  | Validation Set 1 | pT3 | 2 and 3 | 0.244 | 1,239 | 1.29 (0.84–2) |
|  | Validation Set 2 | V1 | 2 and 3 | 0.270 | 738 | 1.2 (0.87–1.65) |
|  | Validation Set 1 | G3 | 2 and 3 | 0.550 | 1,239 | 1.19 (0.68–2.08) |
|  | Validation Set 1 | Age | 2 and 3 | 0.002 | 1,239 | 1.14 (1.05–1.24) |
|  | Validation Set 1 | R1 | 2 and 3 | 0.666 | 1,239 | 1.1 (0.71–1.72) |
|  | Validation Set 1 | G2 | 2 and 3 | 0.897 | 1,239 | 0.96 (0.56–1.66) |
|  | Validation Set 1 | L1 | 2 and 3 | 0.692 | 1,239 | 0.95 (0.74–1.22) |
|  | Validation Set 2 | L1 | 2 and 3 | 0.250 | 738 | 0.92 (0.69–1.23) |
|  | Validation Set 2 | Female sex | 2 and 3 | 0.360 | 738 | 0.89 (0.7–1.14) |
|  | Validation Set 1 | V1 | 2 and 3 | 0.278 | 1,239 | 0.82 (0.57–1.18) |
|  | Validation Set 2 | R1 | 2 and 3 | 0.503 | 738 | 0.81 (0.44–1.5) |
|  | Validation Set 1 | Female sex | 2 and 3 | 0.002 | 1,239 | 0.74 (0.61–0.89) |
| Zhao *et al* [134] |  | Stage III | 1 to 4 | < 0.001 | 315 | 2.98 (1.73–5.15) |
|  |  | Tumour-stroma ratio | 1 to 4 | 0.004 | 315 | 2.08 (1.26–3.42) |
|  |  | Age | 1 to 4 | 0.103 | 315 | 1.02 (1–1.03) |

CI, confidence interval; DFS, disease-free survival; DLS, deep learning system; HR, hazard ratio; L1, presence of lymphovascular invasion. LSTM, long short-term memory; NA, not available; OS, overall survival; Pn1, perineural infiltration; UICC, Union Internationale Contre le Cancer (Union for International Cancer Control); V1, presence of blood vessel infiltration.
